# Notes on Surgical Cases

**Published:** 1860-01

**Authors:** E. Andrews

**Affiliations:** Prof. of Surgery in the Medical Department of Lind University, Chicago


### ORIGINAL COMMUNICATIONS.

### NOTES OF SURGICAL CASES.

BY E. ANDREWS, M. D.,
**Prof. of Surgery in the Medical Department of Lind University, Chicago.**

Case 1. *Fibrous Tumor of the Omentum.* Mrs. X, from ——, applied to me for advice and assistance on account of a large abdominal tumor, of many months standing. It had commenced in one of the iliac regions, and gradually increased to the size of a child's head. The patient at this time was greatly exhausted and suffering from ascites, owing to the irritated state of the peritoneum.

On examination the tumor was found to be exceedingly hard, like a fibrous growth, and nodulated on the surface. It was perfectly moveable, so that it could be rolled to any part of the abdominal cavity, and was entirely free from pain and tenderness. No softness or fluctuation could be detected in any part of it.

On examination, *per vaginum*, I found to my surprise that the tumor had no connection with the uterus; its motions in rolling about having scarcely any effect on the position of that organ.

Having ascertained that the tumor was not uterine, as I at first had supposed, I felt at a loss to determine its true character, for it was decidedly too hard for an ovarian cyst. As the patient obviously could not very long survive the continued dropsy induced by the presence and rapid growth of the abnormal body, it became an important question as to whether its removal should be attempted. I made an appointment for a consultation with some medical gentlemen of the city; but before the hour arrived, the patient's friends suddenly changed their minds and took the cars with her for New York City. On their arrival there, they consulted a gentleman justly eminent in the profession, and holding a prominent chair in one of the Medical Colleges. Whether the parts underwent some change which confused the diagnosis, or whether the patient did not afford the practitioner opportunity for a complete examination, I am not informed, but the result was an error in diagnosis, certainly a very excusable one however under the circumstances. The decision was that the tumor was uterine, and that an attempt at extirpation was hopeless. The patient returned home, and died in about four weeks of chronic peritonitis, manifested by dropsical effusion rather than by the adhesive process.

A post mortem examination was made, which unravelled the mystery. A large fibrous tumor of the omentum was revealed. The uterus was found of the normal size and appearance, and the ovaries healthy. The tumor weighed three pounds and thirteen ounces. The right lung was compressed or atrophied to the size of a closed fist, and the pleural sac filled with a limpid serum. The tumor had some few adhesions by bands to the viscera, but very little vascular supply, and apparently might have been extirpated without difficulty.

*Four cases of Tetanus. Two recoveries.* John B., a negro, received a cut which severed the bone of the thumb, but did not completely divide the soft tissues. Being replaced in position it united well by first intention. On the sixth day he applied for help for stiff jaws, as he called it. On examination he was found to have tetanus. He was ordered ten grains of quinine and half a grain of morphine at one dose, every three hours. For some hours he grew worse, but the enormous doses were continued throughout the night, and the next day he was found greatly improved. Notwithstanding the quantity of quinine taken there was no ringing of the ears, nor did the morphine produce any narcotism. On the second day the intervals between the doses were increased to five hours. The skin which at first had been parched and hot became moist, with a copious warm perspiration, and the articulation regularly improved. The treatment was continued for five days with diminished doses, and the patient was discharged cured. The case was treated by Prof. Johnson, of this city.

Another case, resulting from some injury of the foot, was treated by Prof. W. B. Herrick. All the details of the case, both in symptoms and treatment, corresponded to the previous one, and the cure was equally rapid.

Case third, was that of a man aged forty, who had thrust a nail into his foot. Tetanus supervened, with all the usual symptoms. He was treated by the writer on the narcotic and relaxant plan, the articles used being chiefly cannabis indica and tobacco. Only temporary advantage resulted from this course, and the patient speedily expired from asphyxia, produced by his fearful spasms.

Case four was similar in its origin and course to the third. The patient was treated by Dr. Wardner of this city, by full inhalation of chloroform. This very materially alleviated the suffering, but did not apparently prolong the life. The patient expired of exhaustion at a date as early as he would have done had he been without any treatment.

Tetanus in this region is exceedingly rare, and consequently it becomes practitioners here to discuss it with modesty. The result of the cases treated with narcotics is however in accordance with the general experience of the profession. It is sufficiently settled, that narcotics and antispasmodics are not adequate to the emergency.

It is probable that the spasms derive their uncontrollable violence from the presence of some animal poison, which like that of erysipelas, is generally within the system, and acts directly on the motor nerves, or nerve centres. The attention of the profession should be turned, not to seeking more potent antispasmodics, to resist the effect, but to those remedies known to have the power of destroying animal poisons, with the hope that among them may be found one or more sufficiently prompt and energetic to antidote the cause of all the evil. The sulphate of quinia seems to have been successful in the only two cases in which it was used. Bromine, iodine, the iodides, the chlorates, and perchloride of iron, all have a remarkable power over erysipelas and some other forms of poisonous disease. May they not be efficient in this if given in overwhelming doses?

